# Reovirus and the Host Integrated Stress Response: On the Frontlines of the Battle to Survive

**DOI:** 10.3390/v13020200

**Published:** 2021-01-28

**Authors:** Luke D. Bussiere, Cathy L. Miller

**Affiliations:** Department of Veterinary Microbiology and Preventive Medicine, College of Veterinary Medicine, Iowa State University, Ames, IA 50011, USA; bussiere@iastate.edu

**Keywords:** mammalian orthoreovirus, integrated stress response, eIF2α, translation initiation, stress granules

## Abstract

Cells are continually exposed to stressful events, which are overcome by the activation of a number of genetic pathways. The integrated stress response (ISR) is a large component of the overall cellular response to stress, which ultimately functions through the phosphorylation of the alpha subunit of eukaryotic initiation factor-2 (eIF2α) to inhibit the energy-taxing process of translation. This response is instrumental in the inhibition of viral infection and contributes to evolution in viruses. Mammalian orthoreovirus (MRV), an oncolytic virus that has shown promise in over 30 phase I–III clinical trials, has been shown to induce multiple arms within the ISR pathway, but it successfully evades, modulates, or subverts each cellular attempt to inhibit viral translation. MRV has not yet received Food and Drug Administration (FDA) approval for general use in the clinic; therefore, researchers continue to study virus interactions with host cells to identify circumstances where MRV effectiveness in tumor killing can be improved. In this review, we will discuss the ISR, MRV modulation of the ISR, and discuss ways in which MRV interaction with the ISR may increase the effectiveness of cancer therapeutics whose modes of action are altered by the ISR.

## 1. Introduction

The survival of every organism requires the inheritance of genetic information during reproduction and the ability to adapt to more efficient replication in a changing environment. Since viruses are obligate intracellular parasites, this results in dynamic interactions between viruses and their hosts. Both are subject to the law of natural selection, pitting viruses and their hosts against one another as viruses evolve to better infect and replicate within a host that evolves to more efficiently inhibit viral disease. This struggle between the virus and the host contributes to the evolution we observe in both systems. In animals, simple to complex antiviral responses have been identified that work to recognize and inhibit virus replication and spread, while viruses have evolved counter measures to evade and suppress host defenses. While the immune response is often credited with the full task of protecting animals from viral infection, the role of the cellular stress response, an essential first line of defense that is integral in slowing viral spread and alerting the immune response to impending danger against viral pathogens, is frequently overlooked. In order for viruses to successfully replicate, they need to evade, neutralize, or subvert the stress response.

Mammalian orthoreovirus (MRV) is a double-stranded RNA (dsRNA) virus that belongs to the family *Reoviridae* [1]. The virus contains 10 genome segments that encode eight structural proteins (σ1, σ2, σ3, μ1, μ2, λ1, λ2, and λ3) and four nonstructural proteins (σ1s, σNS, μNS and μNSC) [1]. MRV attaches to cells via junction adhesion molecule-A (JAM-A) and/or sialic acid on the surface of the cell and enters by clathrin-mediated endocytosis [2,3]. Upon entry, the virus outermost capsid proteins are cleaved to activate the viral membrane penetration protein μ1, which pokes holes in the endosome and early lysosome for virus escape [4,5]. The intact viral core enters the cytoplasm and begins transcribing the enclosed dsRNA to produce messenger RNAs (mRNAs) that are then engaged by cellular machinery for primary rounds of translation [6]. Early viral protein synthesis, including that of the factory matrix μNS protein, results in the formation of inclusion bodies, called viral factories (VFs), where subsequent transcription, translation, packaging, replication, and assembly occur [7,8,9,10,11]. MRV is generally considered a clinically benign virus and therefore has been used as a model for the investigation of the structure, function, and host interactions of members of the *Reoviridae* family of viruses for many years. Within the past 20–25 years, MRV has also been investigated as an oncolytic, or cancer-lysing, virus. While MRV was originally thought to primarily kill tumor cells through direct lysis, further investigation in vitro, in preclinical, and in clinical trials has shown that MRV infection of tumor cells elicits an antitumor immune response that is thought to play a major role in the cancer-killing ability of the virus [12,13]. To date, MRV has been investigated in over 30 phase I–III clinical trials against numerous cancers (Table 1) [12,14], and it has shown to be (1) safe, as most reported side effects are considered low-grade, flu-like symptoms, (2) tumor tropic, as most tumors contain replicating MRV in intravenous administered patients, and (3) beneficial to varying degrees to numerous patients with differing cancer types. Additionally, while MRV therapy has been shown to be effective as a monotherapy, almost all phase II and III clinical trials have investigated MRV therapy in combination with standard cancer therapies including chemotherapy, which have been shown to work synergistically with MRV in vitro and in preclinical trials [15,16,17,18]. As MRV has progressed through clinical trials, it has become clear that not all tumors are amenable to MRV therapy, even though the virus is able to infect and replicate in greater than 80% of all tumors when given intravenously [19]. Therefore, it is important to further study virus–host cell interactions to clarify genetic mutations and physiological environments found in tumors that are most amenable to MRV therapy. In this review, we will discuss MRV activation, modulation, and mechanisms employed to evade the cellular integrated stress response (ISR).

## 2. Integrated Stress Response

**Figure 1:**
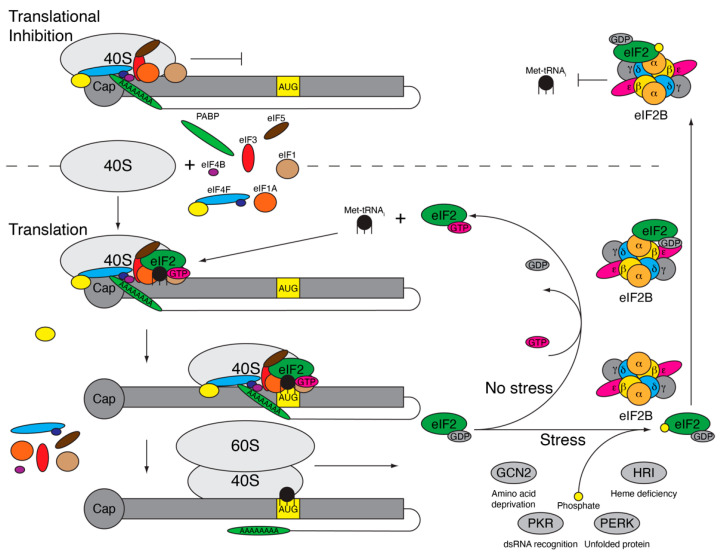
Phosphorylation of eukaryotic initiation factor-2 (eIF2α) results in translational inhibition. Under normal conditions (bottom), the 40S ribosomal subunit and initiation factors eIF1, eIF1A, eIF3, eIF4B, eIF4F complex, and eIF5 come together along with the ternary complex (eIF2, guanosine triphosphate [GTP], and initiator methionine transfer RNA [Met-tRNA_i_}) to form the 48S preinitiation complex (PIC). The 48S PIC begins scanning for the start codon, AUG, in the correct context, resulting in GTP hydrolysis and eIF2–GDP release along with the other initiation factors. The 60S subunit binds with the 40S subunit and translation elongation begins. eIF2–GDP binds eIF2B, which exchanges GDP for GTP, allowing eIF2–GTP to bind another Met-tRNA_i_, form the 48S PIC, and initiate translation again. During stress, eIF2α is phosphorylated by eIF2α kinases (general control non-depressible 2 (GCN2), protein kinase R (PKR), PKR-like endoplasmic reticulum kinase (PERK), or heme-regulated inhibitor (HRI)), triggering eIF2–GDP to get stuck binding eIF2B within the nonproductive site, inhibiting the exchange of GDP for GTP. The 48S PIC, missing ternary complex (48S* PIC), cannot initiate translation and becomes stuck at the 5′ end (top), resulting in ribonucleoprotein complex accumulation and stress granule formation.

The ISR is a cellular process to recognize and respond to various stressors including viral infection, hypoxia, nutrient deficiency, and amino acid deprivation [29,30,31,32]. While the cell utilizes multiple stress pathways to recognize stress including the unfolded protein response (UPR) and the heat shock response (HSR), the ISR is intertwined with these responses and culminates in the inhibition of energy intensive protein translation [33]. When the cell experiences stress, four serine/threonine kinases—heme-regulated inhibitor (HRI), general control non-depressible 2 (GCN2), protein kinase R (PKR), or PKR-like endoplasmic reticulum kinase (PERK)—are activated via dimerization and autophosphorylation [34,35,36,37]. The activated kinases phosphorylate the alpha subunit of eukaryotic initiation factor-2 (eIF2α), resulting in near global translational inhibition as a way for the cell to preserve energy and to direct the translation of select genes involved in assisting with stress mitigation [38]. eIF2α is the regulatory subunit of guanosine triphosphate hydrolase (GTPase) eIF2, which is a heterotrimer composed of eIF2α, eIF2β, and eIF2γ [39]. eIF2 is part of the ternary complex that includes eIF2, GTP, and the initiator methionine transfer RNA (Met-tRNA_i_) and is essential for ribosomal scanning during translation initiation [40]. Before the translation of mRNA begins, the ternary complex binds the 40S ribosomal subunit with initiation factors eIF1, eIF1A, eIF3, and eIF5 to form the 43S preinitiation complex (PIC) (Figure 1) [41]. 43S PIC binds to the mRNA along with the eIF4F complex and eIF4B, at which point it is referred to as the 48S PIC and is primed to begin scanning for the AUG start codon. As the 48S complex moves along the mRNA, the ternary complex is responsible for recognizing the AUG start codon in the appropriate context [42]. Once the AUG is identified, eIF5 functions as a GTPase catalytic protein enhancing eIF2 hydrolysis of bound guanosine triphosphate (GTP) to guanosine diphosphate (GDP) [43]. GTP hydrolysis results in release of the inactive GDP-bound eIF2. Then, eIF5B facilitates the binding of the 60S ribosomal subunit to the 40S ribosomal subunit, and the remaining initiation factors are released [44]. The 80S ribosome is now positioned on the AUG and begins elongation of the protein. Since translation initiation is a cyclic process, each of these proteins and the ribosomal subunits are recycled and can be reutilized for translation initiation. For ternary complex formation, GDP-bound eIF2 must release and replace GDP for GTP, which is a process that occurs slowly without the help of eIF2B—a guanine nucleotide exchange factor (GEF) [45]. eIF2B is made up of two symmetrical and active copies of five subunits: α, β, δ, γ, and ε. While eIF2Bε is the minimal active unit for GEF activity, the efficiency is low and greatly increased upon the full assembly of eIF2B [46]. In addition to eIF2 having a higher affinity for GDP than GTP, eIF5 remains bound to eIF2–GDP and inhibits GDP dissociation to limit the spontaneous release of GDP and binding of GTP [45,47]. The eIF2α subunit facilitates binding to eIF2B into a productive or nonproductive confirmation. eIF2α specifically favors the productive confirmation where the eIF2γ subunit, which is bound to GDP, sits within the eIF2Bε catalytic site [48] and the eIF2α subunit sits within the eIF2Bα/β/δ subunit, allowing the removal of eIF5 and exchange of GTP for GDP-bound eIF2 [46]. GTP-eIF2 is now free to bind Met-tRNA_i_ to form a new ternary complex that is ready to bind the 40S ribosomal subunit and begin ribosomal scanning.

During times of stress, the eIF2α kinases (PKR, PERK, GCN, and HRI) are activated and phosphorylate serine at amino acid 51 (S51) of eIF2α, which alters binding to eIF2B. Phosphorylated (P)-eIF2α is inhibited from binding eIF2B in the productive confirmation and instead binds in a nonproductive confirmation in which eIF2α is bound to the eIF2Bα/β/δ subunit, but within another location, and eIF2γ moves into close proximity with the opposing eIF2Bε subunit (Figure 1) [46]. This not only inhibits the GEF activity of the bound eIF2 protein, but it also prevents the binding of other eIF2 proteins [49]. As eIF2 is expressed in vast excess compared to eIF2B [50], a small increase in the phosphorylation of eIF2α can efficiently inhibit all eIF2B activity. Subsequently, this inhibits GDP removal from eIF2, eIF2–Met–tRNA_i_ binding and the formation of a ternary complex. Therefore, when translation is initiated, the 48S PIC lacking the ternary complex (48S* PIC) stalls at the 5′ end of the mRNA and cannot begin ribosomal scanning [51]. Ribosomes that have already initiated and are undergoing elongation are unaffected and will continue to translate until they fall off the RNA, ultimately resulting in the accumulation of RNAs with one 48S* PIC stalled at the 5′ end. These ribonucleoprotein (RNP) complexes, composed of ribosomal subunits, initiation factors, and mRNA are quickly bound by RNA binding proteins Ras-GAP SH3 domain-binding protein 1 and 2 (G3BP1 and G3BP2), T-cell intracellular antigen 1 (TIA-1), and TIA-1 related protein (TIAR) to form nonmembrane bound inclusions called stress granules (SGs) [52,53]. Stalled RNPs are stored in SGs until the stress within the cell is alleviated.

Even when translation is inhibited, various regulatory processes direct select genes that are necessary to alleviate or control stress to be preferentially translated [54]. While the understanding of mechanisms by which genes are selected to be translated during stress is incomplete, researchers have shown that SGs and upstream open reading frames (uORFs) play key roles in this regulation [55,56,57]. As mentioned above, SGs accumulate stalled RNPs, but they also act as a triage center, allowing specific genes to be translated, degraded, or stored [56]. As SGs form, they are enriched in mRNAs and initiation factors, and it has been suggested that select genes can experience increased translation through the increased formation of initiation complexes in SGs [54,57]. In addition, SGs have also been shown to specifically appropriate large mRNAs, while those that are smaller tend to escape sequestration [58]. This control of release or exemption from sequestration and selective translation may play a role in regulating protein synthesis under stress conditions when SGs are present.

While stress-associated genes are translated preferentially during the stress response, they are often translated at low levels during non-stress conditions. The presence of uORFs ensures that some stress response genes are expressed only when cells are undergoing stress. One of the most studied and best examples of this is within the gene encoding activating transcription factor 4 (ATF4). The ATF4 protein functions as a transcriptional activator that is responsible for the upregulation of many genes, such as C/EBP homologous protein (CHOP) [59], growth arrest and DNA damage 34 (GADD34) [60], eIF4E binding protein 1 (4E-BP1) [61], tRNA synthetases [62], and other proteins that play critical roles in regulating the ISR [63]. The gene for ATF4 has one open reading frame (ORF) that encodes the ATF4 protein, with two uORFs that are non-coding [63]. Under non-stressful conditions, the translational machinery (inclusive of the ribosome and initiation factors) initiates translation at the first uORF and encounters a termination signal; however, the 40S ribosomal subunit reinitiates at the second uORF instead of releasing the mRNA. The second uORF is out of frame with ATF4, and it elongates past the ATF4 encoding ORF, until it encounters a stop codon. This process results in the translation of a non-functional protein. When cells are under stress, the translation initiation factor eIF2α is less available, such that after initiation at the first uORF and termination, the ribosome moves past the second uORF and instead reinitiates at the ATF4 encoding ORF. This combination of initiation/reinitiation events results in the low expression of ATF4 in the absence of stress, and high expression of the protein in the presence of stress [63]. Upon cellular stress, ATF4 and other selectively translated proteins including transcription activators, cell cycle inhibitors, nutrient transporters, tRNA synthetases, and P-eIF2α inhibitors such as GADD34 work together to adapt to the cellular stress and prepare the cell to return to a normal state [64]. If the stress is maintained for an extended time, these proteins instead direct the cell toward autophagy or apoptosis [65].

Together, the ISR works to mitigate the negative impacts of stress on the cell and to return the cell back to normal. It does this through stress sensing by eIF2α kinases, inhibition of ternary complex formation, and SG formation that results in the inhibition of energy-consuming translation. In the context of viral infection-induced translation inhibition, the sequestration of initiation factors to SGs, and induction of autophagy and apoptosis limit the spread of virus throughout the host. Virus-induced stress also inhibits viral spread further via cross talk between the stress response and the innate and adaptive immune response. For example, PKR, P-eIF2α, and ATF4 have been implicated in promoting the production of type-I interferons (IFNs) and the pro-inflammatory interleukin-6 (IL-6) [66]. Therefore, most viruses that successfully infect and replicate within hosts with a stress response have evolved mechanisms to inhibit and/or subvert the ISR.

## 3. Mammalian Orthoreovirus and the ISR

Following binding and endocytosis, MRV is able to escape late endosomes or early lysosomes as early as 1 h post-infection (h p.i.) and begin transcribing and translating protein [67]. Most strains of MRV induce P-eIF2α and SG formation within infected cells by as early as 2 h p.i. [68], which is well before a substantial quantity of viral mRNA can be translated. This poses a problem for the virus, as the inhibition of translation should render the virus inert. However, MRV mRNAs manage to escape this cellular attempt at translational shutoff and continue to be translated. Not only does the virus mRNA escape the translational inhibition of P-eIF2α, but virus-induced cellular stress appears to significantly increase MRV replication [69]. When the active S51 is mutated to an alanine, rendering eIF2α constitutively active in mouse embryonic fibroblasts and preventing translation inhibition through the ISR [70], MRV replicates to lower titers than in cells with phosphorylatable, wild-type eIF2α [69]. Moreover, MRV also replicates to lower titers in ATF4 knockout cells relative to cells with wild-type ATF4 [69]. In addition to MRV benefiting from virus-induced stress, a recent report also suggests that select stressors given prior to virus infection increase MRV replication [71]. Finally, MRV infection itself has been found to induce the expression of ATF4 and downstream effectors of the ISR [69]. This begs the question: how does the virus evade the effects of the ISR, and why would stress be beneficial for the virus?

When MRV infects cells, at least two eIF2α kinases are activated, translation is inhibited, and SGs form within the cell to sequester RNPs [68,69,72]. Virus recognition by the antiviral PKR and ribonuclease L (RNAse L) proteins contribute to translational inhibition observed in most strains of MRV, but at least one additional stress kinase is necessary for the formation of SGs [68,73]. MRV induction of PKR activation is well documented, but the exact mechanism by which the virus induces PKR is unknown. PKR recognizes and becomes active by binding to dsRNA within the cytoplasm [74]. Since dsRNA is a product of replication in RNA viruses, PKR can recognize virus infection and induce translation shutoff. RNAse L is also activated as a result of dsRNA recognition [75]. However, since MRV has been suggested to protect its dsRNA within the viral core throughout infection, it has been difficult to explain the MRV induction of these pathways. More recently, PKR has been shown to recognize structured single-stranded RNA (ssRNA) with dsRNA regions, which might explain its activation during MRV infection [76].

The activation of more than one of the stress kinases is necessary for the formation of SGs, as the deletion of any single kinase is not sufficient to prevent MRV induction of SGs [68]. PKR is likely involved as it has been implicated in activating the ISR as described above. PERK, an endoplasmic reticulum (ER) transmembrane protein that recognizes unfolded protein and ER stress [77], is a second likely candidate stress kinase. MRV has recently been shown to remodel and excise fragments from the ER [78], potentially to act as a platform for viral replication. This remodeling and excising of the ER likely activates PERK. Similar to downstream ISR targets eIF2α and ATF4, PERK knockout leads to a slight inhibition of MRV replication in mouse embryonic fibroblast cells [69], supporting the idea that the virus replicates to increased levels in cells where the ISR is intact. In addition, a recent report found that MRV replicates to higher levels within in vitro models of head and neck cancer in an ATF4-dependent manner in the presence of PERK inhibitor, GSK2606414 [79]. In this same study, while GSK2606414 increased virus replication, PERK knockdown was not beneficial for replication, suggesting that another effect of GSK2606414 is necessary for the observed increase in replication [79]. Together, these findings suggest that an intact and activated ISR, including the downstream effects of P-eIF2α, ATF4, and PERK benefits MRV replication. While less is known about MRV activation of HRI or GCN2, sodium arsenite, a stressor leading to high levels of HRI activation, was shown to increase virus replication when cells were pretreated or treated up to 2 h p.i. [71]. Whether this is dependent on HRI activity is unclear as cellular heat shock, which also activates HRI, does not significantly increase virus replication [71]. As for GCN2, a nonsignificant decrease in ATF4 expression has been shown with GCN2 knockdown in MRV infected cells [79], but further investigation is necessary to determine its relevance.

### 3.1. MRV Evades and Disrupts SGs

Once the ISR is activated, and translation is inhibited by P-eIF2α and SGs, the virus employs various methods to combat this inhibition. First, the virus interferes with SG formation induced by infection. By 2 h p.i. SGs form, and shortly thereafter, viral core particles are observed embedded within SGs [68]. Since viral mRNAs are transcribed from within the core and are released from the λ2 turrets [6], it is possible that the cellular RNA binding proteins found in SGs bind to the exposed mRNA, resulting in viral core sequestration in SGs. This is supported by evidence that transcription is required for the association of the viral core and SGs [68]. Since SGs sort mRNA into groups to be translated, stored, or degraded [56], it stands to reason that SGs may erroneously allow viral mRNA to be translated. SGs are enriched with long mRNAs, on average 7 kB in length, while those that are shorter or have short 3′ untranslated regions (UTRs) are more resistant to SG sequestration [58]. Therefore, one possible mechanism of escape of MRV mRNAs from SGs is that the relatively short MRV mRNAs are not highly enriched at SGs, unlike longer mRNAs. The ten MRV genome segments: small (S) S1–4, medium (M) M1–3, and large (L) L1–3, all produce mRNAs that are smaller than 4 kB long [1], suggesting the virus mRNAs may overcome SG enrichment that other long mRNAs cannot. This may explain why early during infection (2–4 h p.i.), the S (around 1.2 kB) and M (around 2.2 kB) genes express large amounts of protein, while the larger L (around 4 kB) genes express very little [80]. Expression of the L genes at around 4–6 h p.i. correlates well with the timing of SG dissipation [68,80], suggesting that SG disruption may be beneficial for the expression of the L genes.

As infection proceeds, SGs are disrupted, and MRV infection renders cells unable to form SGs, even following treatment with strong stress-inducing drugs [81,82]. The disruption of SGs during MRV infection requires virus gene expression, suggesting that the virus actively prevents SG assembly [68]. While the mechanism by which MRV inhibits SGs is not fully elucidated, SG disruption correlates with the formation of VFs [68], which are inclusion bodies that form during infection where many of the processes of the virus life cycle occur. VFs are primarily composed of the factory matrix protein μNS [7], the RNA binding protein σNS [8], the virus core proteins σ2, μ2, λ1, λ2, and λ3, and the assembled core, and they are the site of viral transcription, translation, packaging, replication, and assembly [7,8,9,10,11]. Early during infection, VFs can be observed in close proximity to SGs [83], and it has been hypothesized that SGs may act as a platform or may contain a cellular protein necessary for formation of VFs. While the SG could act as a platform for VF formation, this does not seem to be a requirement as transfected cells expressing only μNS form viral factory-like (VFL) structures in the absence of SGs [7]. Instead, it seems more plausible that a cellular factor that is necessary for the formation of SGs and VFs is usurped by the virus. This would explain why VF formation coincides with SG disruption and why MRV replicates to higher titers in G3BP1/2 knockout human osteosarcoma U2OS cells, which cannot form SGs, compared to wild-type U2OS cells [82]. Moreover, MRV core VF proteins σNS and μNS are the only viral proteins necessary for SG disruption and inhibition [82]. G3BP1 is a core effector protein necessary for SG assembly that has been shown to associate with σNS [82,84], which may enable MRV disassembly of the SG. During infection, numerous SG-associated proteins including G3BP1/2, cytoplasmic activation- and proliferation-associated protein 1 (caprin1), and ubiquitin-specific protease 10 (USP10) [82], and initiation factors and ribosomal subunits [11] are found around the periphery and within VFs. Sequestering these SG-associated proteins to the VFs may inhibit new SG formation, even in the presence of high levels of P-eIF2α [81]. Altogether, the available data suggests that virus mRNAs evade SG-induced translation inhibition and virus proteins disrupt SG formation, allowing MRV to escape the inhibitory effects of this aspect of the ISR. However, eIF2α phosphorylation is not inhibited in MRV-infected cells [81], even when SGs are disrupted by MRV, and therefore, the virus also requires a mode of escape against the translational inhibition generated by P-eIF2α.

### 3.2. MRV Overcomes the Effects of P-eIF2α

To overcome P-eIF2α so that MRV genes are translated when cellular translation has been effectively shut off as a result of the ISR, the virus must either sequester active ternary complex or use an eIF2-independent method for translation initiation. Several factors necessary for translation initiation are localized to the periphery or within VFs, leading to the hypothesis that the VF acts as a sheltered environment within the cell where viral translation can continue even when there are high levels of P-eIF2α [81]. Viral cores that enter the cell and those that are made during the early stages of infection embed themselves in VFs and transcribe viral mRNA [8]. Initiation factors eIF4E, which binds the 5′ cap, and eIF3A and eIF3B, which make up the 43S PIC, can all be observed within or at the periphery of VFs. Along with 40S and 60S ribosomal subunits and elongation factors, many of the necessary factors are concentrated at the VF, suggesting that they may be exclusively used for MRV specific translation [11,82]. Moreover, ribopuromycylation studies in MRV-infected cells show active translation at the VF, strengthening this premise [11]. While eIF2 has not been shown to be sequestered at the VF, it is possible that eIF2α is selectively and locationally protected from phosphorylation, allowing an active ternary complex to form specifically at the VF, even though global cellular levels of P-eIF2α are high. This hypothesis piggybacks off the observation that the PKR inhibiting MRV protein, σ3, is found localized at the VF in most MRV strains [72]. σ3 binds RNA and inhibits PKR activation and the phosphorylation of eIF2α [85,86]. Thus, PKR is suggested to not be active and eIF2α is not phosphorylated regionally near σ3 localized VFs, whereas PKR remains active outside the VF vicinity elsewhere in the cell. This hypothesis is strengthened when examining σ3 localization in cells infected with the MRV viral strain T3D^F^, which does not induce translation shutoff. The T3D^F^ σ3 protein is diffusely distributed throughout the cytoplasm instead of being localized to the VF as seen in other strains, which is predicted to allow the inhibition of PKR activation throughout the cell [72].

There is strong data supporting that VFs are the sites of viral translation, but the possibility that they can shelter ternary complex protein eIF2 from stress kinases is less certain. The overarching problem with this hypothesis is that MRV induces high levels of P-eIF2α throughout infection while also producing massive amounts of viral proteins [80,81]. As stated above, eIF2 is in vast excess compared to the GEF eIF2B, which is needed for the efficient exchange of GDP with GTP, and P-eIF2α acts as a competitive inhibitor of eIF2B. Therefore, even a slight increase in P-eIF2α results in an almost complete inhibition of eIF2B. Even if VFs can shelter eIF2α from being phosphorylated, once the ternary complex completes its function in translation initiation, eIF2α will not be able to efficiently replace GDP for GTP to become active and to continue initiating new rounds of translation. Thus, for the virus to use eIF2-dependent translation, it seems likely the virus may also employ a mechanism to overcome eIF2B inhibition.

One potential solution to this problem is if the virus encodes or induces an ISR antagonist that can reverse the effects of P-eIF2α inhibition on eIF2B function. Recently, a small molecule ISR inhibitor (ISRIB) was identified that acts to stabilize assembled eIF2B [87,88]. As mentioned previously, eIF2B is composed of two copies of five subunits—α, β, δ, γ, and ε—that assemble to form the fully function eIF2B. ISRIB is able to bind to and stabilize the interaction between the two eIF2Bδ subunits that are part of the regulatory unit composed of eIF2Bα, eIF2Bβ, and eIF2Bδ [88,89]. An increased assembly of eIF2B results in increased GEF activity, allowing for increased resistance to P-eIF2α [90]. If one of the MRV proteins mimics ISRIB, we would expect that even with virus-induced P-eIF2α, the function of eIF2B would be enhanced, and translation could continue. Furthermore, since most of the MRV proteins localize to VF, we might expect this increased eIF2B assembly and function to also localize around the VFs. Another possible solution to this problem would be if MRV encoded a protein that is functionally similar to the AcP10 protein of beluga whale coronavirus, which is a P-eIF2α/eIF2B inhibitor [91]. AcP10 specifically binds to eIF2B and inhibits P-eIF2α binding, thereby allowing non-phosphorylated eIF2α to bind and GEF function to continue, effectively reversing the impact of P-eIF2α even when induced by high levels of stress via sodium arsenite [91]. Further investigation into the function of MRV proteins will identify if the virus encodes proteins involved in altering eIF2B function or has other mechanisms to overcome the eIF2B problem.

### 3.3. MRV May Employ eIF2-Independent and/or Cap-Independent Translation

The above-mentioned hypotheses assume that MRV requires the ternary complex for the translation of its genes, but it is also conceivable, albeit less likely, that MRV uses an eIF2-independent translation initiation [92]. A downstream effect of P-eIF2α is the upregulation of eIF4E binding protein (4E-BP), which is instrumental in the inhibition of cap-dependent translation [93]. Therefore, viruses use internal ribosome entry sequences (IRESs) to recruit the necessary initiation factors and ribosomal subunits independent of the 5′ cap. One class of IRESs, class 4, are the only IRESs to date that have been identified to also be independent of eIF2 [94]. The identified class 4 IRESs are 150–200 nucleotide long RNA structures between two ORFs that bind and recruit the 40S ribosomal subunit to the AUG start codon where the 80S ribosome assembles and translation begins [95]. Interestingly, IRESs encoded in viruses of the *Dicistroviridae* family have been shown to benefit from cellular stress similar to MRV [96], but this seems to be a rare commonality between MRV and the *Dicistroviridae*. MRV mRNAs have not been demonstrated to possess IRESs, and the 5′ UTR proceeding the AUG start codon of each gene segment is likely too short to form an IRES. Another method of eIF2-independent translation initiation that may be more plausible for MRV is the use of a different initiation factor than eIF2 for introduction of the Met-tRNA_i_. Both hepatitis C virus [97] and poliovirus [98] have been suggested to use eIF5B to deliver Met-tRNA_i_ in the absence of eIF2 when levels of P-eIF2α are high. While eIF5B has not historically been recognized for its ability to bring the Met-tRNA_i_ to the 43S PIC, it is a homolog of the prokaryotic IF2 [99], which is part of the ternary complex and is responsible for delivering the Met-tRNA_i_ in prokaryotes similar to eIF2 in eukaryotes [100]. As more research has detailed the function of eIF5B, it is now clear that viruses as well as cellular genes (i.e., X-linked inhibitor of apoptosis protein [XIAP]) can utilize eIF5B instead of eIF2 during cellular stress [101]. Thus far, eIF5B has not been formally identified at VFs, but as one of the SG-associated proteins, it is plausible that SG disruption and recruitment could result in eIF5B localization to VFs.

An additional hurdle MRV must overcome to continue viral protein translation in the stressed cell is stress-induced inhibition of cap-dependent translation. During prolonged exposure of stress and P-eIF2α, cells halt cap-dependent translation in favor of cap-independent translation [102]. ATF4, which is selectively translated during stress via an uORF mechanism discussed above, upregulates 4E-BP [93], which accumulates and competitively binds eIF4E to inhibit binding to the 5′ cap. Stress-associated proteins including hypoxia-inducible factor 1α (HIF-1α), fibroblast growth factor 9 (FGF-9), and p53 possess mechanisms to overcome this inhibition [103]. This is accomplished by utilizing death associated protein 5 (DAP5) and eIF4GI, both of which are suggested to be upregulated during stress [104,105], bind RNA structures within the 5′ UTR, and restore translation in a cap-independent manner [103]. DAP5 and eIF4GI are homologs and associate with additional initiation factors, suggesting that DAP5 or eIF4GI binding recruits the necessary factors for translation initiation. The use of DAP5, eIF4GI, or a similar factor by MRV to overcome cap-dependent translation inhibition is an intriguing hypothesis, as the virus has been suggested to switch from cap-dependent translation early during infection to cap-independent translation as infection proceeds [106]. Furthermore, MRV has been shown to effectively inhibit translation of the stress protein HIF-1α [107,108], which may suggest that MRV can usurp DAP5 or eIF4GI for viral protein translation, thereby inhibiting HIF-1α expression. Whether MRV mRNAs have the necessary structure within the 5′ UTR for DAP5 or eIF4GI recognition remains to be determined.

### 3.4. MRV Benefits from the ISR

**Figure 2:**
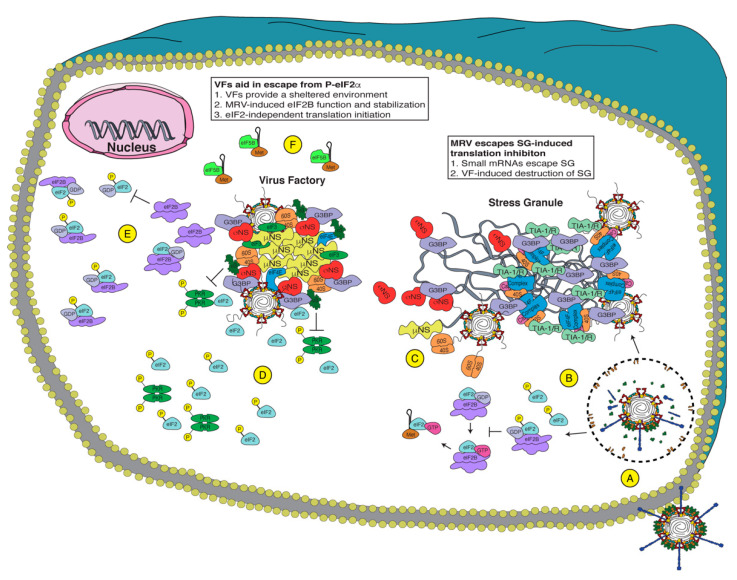
Potential mechanisms of Mammalian orthoreovirus (MRV) evasion of the integrated stress response (ISR). Upon virus attachment (**A**), MRV is endocytosed and escapes the endosome/lysosome to release the transcribing core. Early during infection (**B**), MRV induces P-eIF2α, inhibiting eIF2B guanine nucleotide exchange factor (GEF) activity, shutting off translation, and inducing stress granule (SG) formation. Since MRV encodes small messenger RNAs (mRNAs) (**C**), the viral mRNAs escape the SG and are translated, allowing viral factories (VFs) formation, which disrupt SGs. The virus efficiently translates its mRNA even with high levels of P-eIF2α, and therefore, it uses one or more of these methods: (**D**) VFs act as a shelter to inhibit eIF2α phosphorylation and sequester necessary components for translation to the VF, (**E**) MRV encodes a protein that inhibits P-eIF2α binding to eIF2B and/or stabilizes the fully assembled eIF2B, or (**F**) MRV uses eIF2-independent translation, employing other initiation factors (i.e., eIF5B) to deliver Met-tRNA_i_. Together, MRV uses these methods to allow the translation of viral proteins when cellular translation is inhibited.

Regardless of the mechanism in which MRV overcomes the individual hurdles that result from the ISR, such as P-eIF2α, SGs, and subsequent translational inhibition (Figure 2), the virus not only successfully evades the ISR but appears to benefit from its activation. This suggests that the cellular environment presented during the ISR awards the virus with conditions that lead to more efficient replication. Since the stress response is intimately connected with modifying and relocalizing the cellular translational machinery, this suggests that the MRV benefit from stress induction may be a result of the virus taking advantage of these altered regulatory processes involved in mRNA translation. When the cell is alerted to viral infection via PKR and other stress kinases, large amounts of translation factors are sequestered at SGs [109]. This may organize the translation initiation factors into a confined space that is ripe for virus commandeering and exploitation, giving the virus an advantage in replication relative to non-stressed situations. Supporting this hypothesis, MRV localizes to and disrupts SGs early during infection, freeing the translation initiation factors from SG sequestration and recruiting them to VFs for use in viral mRNA translation. Moreover, following SG disruption, the virus may continue to benefit from the ISR, as MRV mRNA can exclusively be translated in the presence of increased levels of P-eIF2α, which inhibits a majority of the competing cellular mRNA translation. This may stem from as yet incompletely described methods used by MRV to locationally inhibit or overcome P-eIF2α and eIF2B at the VF. However, SG disruption, VF localization of and regulation of P-eIF2α/eIF2B levels, and overcoming translational shutoff does not tell the entire story, as MRV also replicates better in cells where ATF4, a downstream ISR gene, can be activated. This is not an MRV-specific phenomena, as human immunodeficiency virus (HIV) and murine cytomegalovirus (MCMV) also both benefit from ATF4 signaling [110,111]. In addition, research suggests that ATF4 is an inhibitor of interferon regulatory factor 7 (IRF7), which is a main regulator of type-I IFN [112]. IRF7 is normally expressed at baseline levels but is greatly increased during virus infection [112], suggesting that ATF4 activation may decrease the negative impacts of type-I IFN on virus replication. MRV may also benefit from actions present in ATF4-regulated genes, such as GADD34-induced inhibition of eIF2α phosphorylation [60,113], or by other, as yet unidentified processes. Therefore, while it makes sense that MRV would benefit from the ISR as many cellular factors necessary for translation are organized into SGs and usurped specifically for MRV translation, there is still much to be learned about MRV and its interaction with the ISR.

## 4. Mammalian Orthoreovirus Modulation of the ISR Impact on Cancer Therapeutics

Cancer causes the second most deaths globally each year behind cardiovascular diseases. While cancer can be classified by the site in the body, the tissue type, and the genetic markers it expresses, each patient’s cancer is completely distinct and varies compared to other patients [114]. This makes treating cancer patients difficult even with a wide gamut of cancer therapeutics available and has shuttled in a new era of personalized medicine that is tailored to each person [115]. It is becoming commonplace for clinicians to biopsy a patient’s tumor for sequencing, aiding in the determination of the appropriate therapy for that individual’s tumor [116]. Even with our advances in cancer treatment, it can be difficult to successfully treat advanced tumors that are resistant to many standard therapies. A large driver of tumor resistance to therapy is the cellular stress response, which has been shown to confer resistance to chemotherapy and radiotherapy (Table 1) [117]. Both of these therapies also induce large amounts of cellular stress exacerbating this problem, and therefore, novel therapeutics that inhibit or overcome the ISR are needed to combat ISR-induced resistance. As mentioned previously, MRV has been shown to work synergistically with various therapeutic agents in vitro and in preclinical trials including radiation, docetaxel, rapamycin, paclitaxel, gemcitabine, fluorouracil, cisplatin, and doxorubicin [15,16,17,18]. It is interesting to speculate that MRV modulation of the cellular stress response may play a role in this synergy.

While the ISR is meant to bring stressed cells back into homeostasis, this can be an issue in cancers, as the stress response is modified in many tumors, leading to resistance to various therapies. For instance, in 15% of prostate cancer patients, a mutated SPOP gene results in excess caprin1 expression, resulting in increased SG formation [26]. This is problematic, as SGs mitigate the effect of docetaxel, which is the most common drug administered to prostate cancer patients [26]. Another chemotherapeutic that is sensitive to SGs is oxaliplatin, which is a common treatment for colon cancer. Oxaliplatin has been shown to be ineffective in Kirsten rat sarcoma 2 viral oncogene (KRAS)-mutated colon cancers where SGs are prevalent. When SGs are inhibited with cyclooxygenase 1/2 (COX1/2) inhibitors in KRAS-mutated colon cancers, researchers found that the cells were resensitized to oxaliplatin [118]. SGs can also make tumors more aggressive. For example, the SG-associating protein receptor for activated C kinase 1 (RACK1) is sequestered to SGs during stress, inhibiting its apoptotic induction. Morusin, a novel drug from the *Morus alba linn* root, has been shown to use RACK1-dependent apoptosis to induce cancer cell killing in vitro, but it also induces SGs [119]. When morusin was used with ISRIB to inhibit SG formation, there was significantly more cell killing [119], suggesting that SG disruption, a well-defined phenotype of MRV infection, benefits the activity of the drug. In addition, when G3BP1, the critical SG effector protein, is knocked down in osteosarcoma cells and then implanted in mice, there are decreased SGs and metastasis compared to wild-type cells [120]. Together, these studies suggest that SG disruption can benefit cancer patients by inhibiting tumor aggressiveness, spread, and resistance to therapy.

Apart from the well-documented undesirable impact of SGs, researchers have identified other aspects of the ISR that negatively impact cancer prognosis and treatment. In acute myeloid leukemia patients, repeat exposure to daunorubicin has been shown to induce the ISR and ATF4 expression, resulting in increased drug efflux, via ATP binding cassette subfamily B member 1 (ABCB1), and treatment failure [121]. When ISRIB and U0126, a MAPK/ERK kinase 1/2 (MEK1/2) inhibitor, were used to inhibit stress signaling, daunorubicin-resistant K562 bone marrow cells were resensitized to drug [121]. In addition, gemcitabine-resistant pancreatic ductal adenocarcinoma has increased gemcitabine-mediated apoptosis when ISRIB or siRNA against ATF4 is introduced to cells [20]. Realizing the impact of the ISR on cancer patients, researchers now suggest that stress markers P-eIF2α and PKR can be used as biomarkers to identify cancers with increased cellular stress and as a tool for patient prognosis [122,123,124]. Taken altogether, these examples confirm the need for novel therapeutics that specifically target the ISR and SGs and based on our understanding of MRV disruption of SGs, enhanced replication in stressed cells, and past clinical successes suggest that MRV may be an ideal candidate therapeutic for tackling stress resistance in cancer therapy.

## 5. Conclusions

The ISR is an important regulator of cellular stress and an important blockade for the invasion of viruses. Upon MRV infection, virus detection by eIF2α kinases, phosphorylation of eIF2α, inhibition of translation, and sequestration of translation machinery to SGs work together to prevent virus translation and replication. Unfortunately for the cell, MRV has evolved various mechanisms to evade, modulate, and subvert the ISR to successfully replicate within the cell. Since MRV is an oncolytic virus that has been shown to work synergistically with various cancer therapeutics that are altered by the ISR, this points to MRV as a potentially ideal supplementing therapeutic to use in concert with these drugs. Further investigation into the battle between MRV and the ISR will increase our understanding of the basic biology of the virus and likely identify possible applications for MRV therapy as a synergistic treatment for tumors undergoing stress, either from mutation, environment, or as a result of other therapeutic approaches.

## Figures and Tables

**Table 1:** Summary of current (black), completed (green), and withdrawn (red) clinical trials listed on www.clinicaltrials.gov.

Cancer	NCT Number	Phase	Treatment	Specifically Inhibited by ISR
Bladder	NCT02723838	I	Pelareorep/Reolysin/Reovirus Gemcitabine Cisplatin	Gemcitabine: [20] Cisplatin: [21,22]
Bone and soft tissue	NCT00503295	II	Pelareorep/Reolysin/Reovirus	
Brain	NCT00528684	I	Pelareorep/Reolysin/Reovirus	
	NCT02444546	I	Pelareorep/Reolysin/Reovirus GM-CSF	
Breast	NCT01656538	II	Pelareorep/Reolysin/Reovirus Paclitaxel	Paclitaxel: [23]
	NCT04102618	I	Pelareorep/Reolysin/Reovirus Letrozole Atezolizumab Trastuzumab	
	NCT04445844	II	Pelareorep/Reolysin/Reovirus Retifanlimab	
	NCT04215146	II	Pelareorep/Reolysin/Reovirus Avelumab Paclitaxel	Paclitaxel: [23]
Colorectal	NCT01274624	I	Pelareorep/Reolysin/Reovirus Irinotecan Leucovorin Fluorouracil Bevacizumab	Fluorouracil: [24]
	NCT01622543	II	Pelareorep/Reolysin/Reovirus Folfox Bevacizumab	
Head and neck	NCT00753038	II	Pelareorep/Reolysin/Reovirus Carboplatin Paclitaxel	Paclitaxel: [23]
	NCT01166542	III	Pelareorep/Reolysin/Reovirus Carboplatin Paclitaxel	Paclitaxel: [23]
Lung	NCT01708993	II	Pelareorep/Reolysin/Reovirus Pemetrexed Docetaxel	Pemetrexed: [25] Docetaxel: [26]
	NCT00861627	II	Pelareorep/Reolysin/Reovirus Carboplatin Paclitaxel	Paclitaxel: [23]
	NCT00998192	II	Pelareorep/Reolysin/Reovirus Paclitaxel Carboplatin	Paclitaxel: [23]
Melanoma	NCT00984464	II	Pelareorep/Reolysin/Reovirus Carboplatin Paclitaxel	Paclitaxel: [23]
	NCT00651157	II	Pelareorep/Reolysin/Reovirus	
	NCT03282188	I/II	Pelareorep/Reolysin/Reovirus GM-CSF	
Myeloma	NCT01533194	I	Pelareorep/Reolysin/Reovirus	
	NCT02514382	I	Pelareorep/Reolysin/Reovirus Bortezomib Dexamethasone	Bortezomib: [27,28]
	NCT03015922	I	Pelareorep/Reolysin/Reovirus Lenalidomide Pomalidomide	
	NCT02101944	I	Pelareorep/Reolysin/Reovirus Carfilzomib Dexamethasone	
	NCT03605719	I	Pelareorep/Reolysin/Reovirus Carfilzomib Dexamethasone Nivolumab	
Ovarian or fallopian tube	NCT00602277	I	Pelareorep/Reolysin/Reovirus	
	NCT01199263	II	Pelareorep/Reolysin/Reovirus Paclitaxel	Paclitaxel: [23]
Pancreatic	NCT02620423	I	Pelareorep/Reolysin/Reovirus Gemcitabine Irinotecan Leucovorin Fluorouracil Pembrolizumab	Gemcitabine: [20] Fluorouracil: [24]
	NCT00998322	II	Pelareorep/Reolysin/Reovirus Gemcitabine	
	NCT01280058	II	Pelareorep/Reolysin/Reovirus Carboplatin Paclitaxel	Paclitaxel: [23]
	NCT03723915	II	Pelareorep/Reolysin/Reovirus Pembrolizumab	
Prostate	NCT01619813	II	Pelareorep/Reolysin/Reovirus Docetaxel, Prednisone	Docetaxel: [26]
Unspecified	NCT01240538	I	Pelareorep/Reolysin/Reovirus Cyclophosphamide	

## References

[1] Knipe D.M., Howley P.M. (2001). Fundamental Virology.

[2] Barton E.S., Forrest J.C., Connolly J.L., Chappell J.D., Liu Y., Schnell F.J., Nusrat A., Parkos C.A., Dermody T.S. (2001). Junction adhesion molecule is a receptor for reovirus. Cell.

[3] Chappell J.D., Gunn V.L., Wetzel J.D., Baer G.S., Dermody T.S. (1997). Mutations in type 3 reovirus that determine binding to sialic acid are contained in the fibrous tail domain of viral attachment protein sigma1. J. Virol..

[4] Nibert M.L., Schiff L.A., Fields B.N. (1991). Mammalian reoviruses contain a myristoylated structural protein. J. Virol..

[5] Agosto M.A., Ivanovic T., Nibert M.L. (2006). Mammalian reovirus, a nonfusogenic nonenveloped virus, forms size-selective pores in a model membrane. Proc. Natl. Acad. Sci. USA.

[6] Coombs K.M. (2006). Reovirus structure and morphogenesis. Curr. Top. Microbiol. Immunol..

[7] Broering T.J., Parker J.S., Joyce P.L., Kim J., Nibert M.L. (2002). Mammalian reovirus nonstructural protein microNS forms large inclusions and colocalizes with reovirus microtubule-associated protein micro2 in transfected cells. J. Virol..

[8] Broering T.J., Kim J., Miller C.L., Piggott C.D., Dinoso J.B., Nibert M.L., Parker J.S. (2004). Reovirus nonstructural protein mu NS recruits viral core surface proteins and entering core particles to factory-like inclusions. J. Virol..

[9] Miller C.L., Broering T.J., Parker J.S., Arnold M.M., Nibert M.L. (2003). Reovirus sigma NS protein localizes to inclusions through an association requiring the mu NS amino terminus. J. Virol..

[10] Miller C.L., Arnold M.M., Broering T.J., Eichwald C., Kim J., Dinoso J.B., Nibert M.L. (2007). Virus-derived platforms for visualizing protein associations inside cells. Mol. Cell. Proteom..

[11] Desmet E.A., Anguish L.J., Parker J.S. (2014). Virus-mediated compartmentalization of the host translational machinery. mBio.

[12] Gong J., Sachdev E., Mita A.C., Mita M.M. (2016). Clinical development of reovirus for cancer therapy: An oncolytic virus with immune-mediated antitumor activity. World J. Methodol..

[13] Müller L., Berkeley R., Barr T., Ilett E., Errington-Mais F. (2020). Past, Present and Future of Oncolytic Reovirus. Cancers.

[14] Phillips M.B., Stuart J.D., Rodríguez Stewart R.M., Berry J.T., Mainou B.A., Boehme K.W. (2018). Current understanding of reovirus oncolysis mechanisms. Oncolytic Virother..

[15] Comins C., Simpson G.R., Rogers W., Relph K., Harrington K., Melcher A., Roulstone V., Kyula J., Pandha H. (2018). Synergistic antitumour effects of rapamycin and oncolytic reovirus. Cancer Gene Ther..

[16] Heinemann L., Simpson G.R., Boxall A., Kottke T., Relph K.L., Vile R., Melcher A., Prestwich R., Harrington K.J., Morgan R. (2011). Synergistic effects of oncolytic reovirus and docetaxel chemotherapy in prostate cancer. BMC Cancer.

[17] Roulstone V., Twigger K., Zaidi S., Pencavel T., Kyula J.N., White C., McLaughlin M., Seth R., Karapanagiotou E.M., Mansfield D. (2013). Synergistic cytotoxicity of oncolytic reovirus in combination with cisplatin-paclitaxel doublet chemotherapy. Gene Ther..

[18] Maitra R., Ghalib M.H., Goel S. (2012). Reovirus: A targeted therapeutic--progress and potential. Mol. Cancer Res..

[19] Moore M., McCarthy T., Spark J. Oncolytics Biotech^®^ Presents Clinical Data Highlighting the Effectiveness of Intravenous Delivery to and Replication of Pelareorep in Tumors.

[20] Palam L.R., Gore J., Craven K.E., Wilson J.L., Korc M. (2015). Integrated stress response is critical for gemcitabine resistance in pancreatic ductal adenocarcinoma. Cell Death Dis..

[21] Wang S.F., Wung C.H., Chen M.S., Chen C.F., Yin P.H., Yeh T.S., Chang Y.L., Chou Y.C., Hung H.H., Lee H.C. (2018). Activated Integrated Stress Response Induced by Salubrinal Promotes Cisplatin Resistance in Human Gastric Cancer Cells via Enhanced xCT Expression and Glutathione Biosynthesis. Int. J. Mol. Sci..

[22] Zhang H., Zhang S., He H., Zhao W., Chen J., Shao R.G. (2012). GAP161 targets and downregulates G3BP to suppress cell growth and potentiate cisplaitin-mediated cytotoxicity to colon carcinoma HCT116 cells. Cancer Sci..

[23] Chen L., He J., Zhou J., Xiao Z., Ding N., Duan Y., Li W., Sun L.Q. (2019). EIF2A promotes cell survival during paclitaxel treatment in vitro and in vivo. J. Cell. Mol. Med..

[24] Shi Z., Yu X., Yuan M., Lv W., Feng T., Bai R., Zhong H. (2019). Activation of the PERK-ATF4 pathway promotes chemo-resistance in colon cancer cells. Sci. Rep..

[25] Reich S., Nguyen C.D.L., Has C., Steltgens S., Soni H., Coman C., Freyberg M., Bichler A., Seifert N., Conrad D. (2020). A multi-omics analysis reveals the unfolded protein response regulon and stress-induced resistance to folate-based antimetabolites. Nat. Commun..

[26] Shi Q., Zhu Y., Ma J., Chang K., Ding D., Bai Y., Gao K., Zhang P., Mo R., Feng K. (2019). Prostate Cancer-associated SPOP mutations enhance cancer cell survival and docetaxel resistance by upregulating Caprin1-dependent stress granule assembly. Mol. Cancer.

[27] Fournier M.J., Gareau C., Mazroui R. (2010). The chemotherapeutic agent bortezomib induces the formation of stress granules. Cancer Cell Int..

[28] White M.C., Schroeder R.D., Zhu K., Xiong K., McConkey D.J. (2018). HRI-mediated translational repression reduces proteotoxicity and sensitivity to bortezomib in human pancreatic cancer cells. Oncogene.

[29] Harding H.P., Zhang Y., Zeng H., Novoa I., Lu P.D., Calfon M., Sadri N., Yun C., Popko B., Paules R. (2003). An integrated stress response regulates amino acid metabolism and resistance to oxidative stress. Mol. Cell.

[30] Levin D., London I.M. (1978). Regulation of protein synthesis: Activation by double-stranded RNA of a protein kinase that phosphorylates eukaryotic initiation factor 2. Proc. Natl. Acad. Sci. USA.

[31] Rzymski T., Milani M., Pike L., Buffa F., Mellor H.R., Winchester L., Pires I., Hammond E., Ragoussis I., Harris A.L. (2010). Regulation of autophagy by ATF4 in response to severe hypoxia. Oncogene.

[32] Ye J., Kumanova M., Hart L.S., Sloane K., Zhang H., De Panis D.N., Bobrovnikova-Marjon E., Diehl J.A., Ron D., Koumenis C. (2010). The GCN2-ATF4 pathway is critical for tumour cell survival and proliferation in response to nutrient deprivation. EMBO J..

[33] Costa-Mattioli M., Walter P. (2020). The integrated stress response: From mechanism to disease. Science.

[34] Bauer B.N., Rafie-Kolpin M., Lu L., Han A., Chen J.J. (2001). Multiple autophosphorylation is essential for the formation of the active and stable homodimer of heme-regulated eIF2alpha kinase. Biochemistry.

[35] Liu C.Y., Schröder M., Kaufman R.J. (2000). Ligand-independent dimerization activates the stress response kinases IRE1 and PERK in the lumen of the endoplasmic reticulum. J. Biol. Chem..

[36] Lemaire P.A., Lary J., Cole J.L. (2005). Mechanism of PKR activation: Dimerization and kinase activation in the absence of double-stranded RNA. J. Mol. Biol..

[37] Narasimhan J., Staschke K.A., Wek R.C. (2004). Dimerization is required for activation of eIF2 kinase Gcn2 in response to diverse environmental stress conditions. J. Biol. Chem..

[38] Taniuchi S., Miyake M., Tsugawa K., Oyadomari M., Oyadomari S. (2016). Integrated stress response of vertebrates is regulated by four eIF2α kinases. Sci. Rep..

[39] Kimball S.R. (1999). Eukaryotic initiation factor eIF2. Int. J. Biochem. Cell Biol..

[40] Levin D.H., Kyner D., Acs G. (1973). Protein initiation in eukaryotes: Formation and function of a ternary complex composed of a partially purified ribosomal factor, methionyl transfer RNA, and guanosine triphosphate. Proc. Natl. Acad. Sci. USA.

[41] Jackson R.J., Hellen C.U., Pestova T.V. (2010). The mechanism of eukaryotic translation initiation and principles of its regulation. Nat. Rev. Mol. Cell Biol..

[42] Hernández G., Osnaya V.G., Pérez-Martínez X. (2019). Conservation and Variability of the AUG Initiation Codon Context in Eukaryotes. Trends Biochem. Sci..

[43] Paulin F.E., Campbell L.E., O’Brien K., Loughlin J., Proud C.G. (2001). Eukaryotic translation initiation factor 5 (eIF5) acts as a classical GTPase-activator protein. Curr. Biol..

[44] Pestova T.V., Lomakin I.B., Lee J.H., Choi S.K., Dever T.E., Hellen C.U. (2000). The joining of ribosomal subunits in eukaryotes requires eIF5B. Nature.

[45] Singh C.R., Lee B., Udagawa T., Mohammad-Qureshi S.S., Yamamoto Y., Pavitt G.D., Asano K. (2006). An eIF5/eIF2 complex antagonizes guanine nucleotide exchange by eIF2B during translation initiation. EMBO J..

[46] Kashiwagi K., Yokoyama T., Nishimoto M., Takahashi M., Sakamoto A., Yonemochi M., Shirouzu M., Ito T. (2019). Structural basis for eIF2B inhibition in integrated stress response. Science.

[47] Jennings M.D., Pavitt G.D. (2010). eIF5 has GDI activity necessary for translational control by eIF2 phosphorylation. Nature.

[48] Fabian J.R., Kimball S.R., Heinzinger N.K., Jefferson L.S. (1997). Subunit assembly and guanine nucleotide exchange activity of eukaryotic initiation factor-2B expressed in Sf9 cells. J. Biol. Chem..

[49] Rowlands A.G., Panniers R., Henshaw E.C. (1988). The catalytic mechanism of guanine nucleotide exchange factor action and competitive inhibition by phosphorylated eukaryotic initiation factor 2. J. Biol. Chem..

[50] Singh C.R., Udagawa T., Lee B., Wassink S., He H., Yamamoto Y., Anderson J.T., Pavitt G.D., Asano K. (2007). Change in nutritional status modulates the abundance of critical pre-initiation intermediate complexes during translation initiation in vivo. J. Mol. Biol..

[51] Kedersha N., Chen S., Gilks N., Li W., Miller I.J., Stahl J., Anderson P. (2002). Evidence that ternary complex (eIF2-GTP-tRNA(i)(Met))-deficient preinitiation complexes are core constituents of mammalian stress granules. Mol. Biol. Cell.

[52] Kedersha N.L., Gupta M., Li W., Miller I., Anderson P. (1999). RNA-binding proteins TIA-1 and TIAR link the phosphorylation of eIF-2 alpha to the assembly of mammalian stress granules. J. Cell Biol..

[53] Matsuki H., Takahashi M., Higuchi M., Makokha G.N., Oie M., Fujii M. (2013). Both G3BP1 and G3BP2 contribute to stress granule formation. Genes Cells.

[54] Buchan J.R., Parker R. (2009). Eukaryotic stress granules: The ins and outs of translation. Mol. Cell.

[55] Andreev D.E., O’Connor P.B., Fahey C., Kenny E.M., Terenin I.M., Dmitriev S.E., Cormican P., Morris D.W., Shatsky I.N., Baranov P.V. (2015). Translation of 5’ leaders is pervasive in genes resistant to eIF2 repression. Elife.

[56] Kedersha N., Anderson P. (2002). Stress granules: Sites of mRNA triage that regulate mRNA stability and translatability. Biochem. Soc. Trans..

[57] Balagopal V., Parker R. (2009). Polysomes, P bodies and stress granules: States and fates of eukaryotic mRNAs. Curr. Opin Cell Biol..

[58] Khong A., Matheny T., Jain S., Mitchell S.F., Wheeler J.R., Parker R. (2017). The Stress Granule Transcriptome Reveals Principles of mRNA Accumulation in Stress Granules. Mol. Cell.

[59] Fawcett T.W., Martindale J.L., Guyton K.Z., Hai T., Holbrook N.J. (1999). Complexes containing activating transcription factor (ATF)/cAMP-responsive-element-binding protein (CREB) interact with the CCAAT/enhancer-binding protein (C/EBP)-ATF composite site to regulate Gadd153 expression during the stress response. Biochem. J..

[60] Novoa I., Zeng H., Harding H.P., Ron D. (2001). Feedback inhibition of the unfolded protein response by GADD34-mediated dephosphorylation of eIF2alpha. J. Cell Biol..

[61] Vasudevan D., Clark N.K., Sam J., Cotham V.C., Ueberheide B., Marr M.T., Ryoo H.D. (2017). The GCN2-ATF4 Signaling Pathway Induces 4E-BP to Bias Translation and Boost Antimicrobial Peptide Synthesis in Response to Bacterial Infection. Cell Rep..

[62] Chen H., Pan Y.X., Dudenhausen E.E., Kilberg M.S. (2004). Amino acid deprivation induces the transcription rate of the human asparagine synthetase gene through a timed program of expression and promoter binding of nutrient-responsive basic region/leucine zipper transcription factors as well as localized histone acetylation. J. Biol. Chem..

[63] Kilberg M.S., Shan J., Su N. (2009). ATF4-dependent transcription mediates signaling of amino acid limitation. Trends Endocrinol. Metab..

[64] Pakos-Zebrucka K., Koryga I., Mnich K., Ljujic M., Samali A., Gorman A.M. (2016). The integrated stress response. EMBO Rep..

[65] Wang C., Tan Z., Niu B., Tsang K.Y., Tai A., Chan W.C.W., Lo R.L.K., Leung K.K.H., Dung N.W.F., Itoh N. (2018). Inhibiting the integrated stress response pathway prevents aberrant chondrocyte differentiation thereby alleviating chondrodysplasia. Elife.

[66] Cláudio N., Dalet A., Gatti E., Pierre P. (2013). Mapping the crossroads of immune activation and cellular stress response pathways. EMBO J..

[67] Kounatidis I., Stanifer M.L., Phillips M.A., Paul-Gilloteaux P., Heiligenstein X., Wang H., Okolo C.A., Fish T.M., Spink M.C., Stuart D.I. (2020). 3D Correlative Cryo-Structured Illumination Fluorescence and Soft X-ray Microscopy Elucidates Reovirus Intracellular Release Pathway. Cell.

[68] Qin Q., Hastings C., Miller C.L. (2009). Mammalian orthoreovirus particles induce and are recruited into stress granules at early times postinfection. J. Virol..

[69] Smith J.A., Schmechel S.C., Raghavan A., Abelson M., Reilly C., Katze M.G., Kaufman R.J., Bohjanen P.R., Schiff L.A. (2006). Reovirus induces and benefits from an integrated cellular stress response. J. Virol..

[70] Scheuner D., Song B., McEwen E., Liu C., Laybutt R., Gillespie P., Saunders T., Bonner-Weir S., Kaufman R.J. (2001). Translational control is required for the unfolded protein response and in vivo glucose homeostasis. Mol. Cell.

[71] Lutz M.M., Worth M.P., Hinchman M.M., Parker J.S.L., Ledgerwood E.D. (2019). Infection is Enhanced in Cells Pre-Treated with Sodium Arsenite. Viruses.

[72] Schmechel S., Chute M., Skinner P., Anderson R., Schiff L. (1997). Preferential translation of reovirus mRNA by a sigma3-dependent mechanism. Virology.

[73] Smith J.A., Schmechel S.C., Williams B.R., Silverman R.H., Schiff L.A. (2005). Involvement of the interferon-regulated antiviral proteins PKR and RNase L in reovirus-induced shutoff of cellular translation. J. Virol..

[74] Feng G.S., Chong K., Kumar A., Williams B.R. (1992). Identification of double-stranded RNA-binding domains in the interferon-induced double-stranded RNA-activated p68 kinase. Proc. Natl. Acad. Sci. USA.

[75] Clemens M.J., Williams B.R. (1978). Inhibition of cell-free protein synthesis by pppA2’p5’A2’p5’A: A novel oligonucleotide synthesized by interferon-treated L cell extracts. Cell.

[76] Mayo C.B., Cole J.L. (2017). Interaction of PKR with single-stranded RNA. Sci. Rep..

[77] Harding H.P., Zhang Y., Ron D. (1999). Protein translation and folding are coupled by an endoplasmic-reticulum-resident kinase. Nature.

[78] Tenorio R., de Castro I.F., Knowlton J.J., Zamora P.F., Lee C.H., Mainou B.A., Dermody T.S., Risco C. (2018). Reovirus σNS and μNS Proteins Remodel the Endoplasmic Reticulum to Build Replication Neo-Organelles. mBio.

[79] McLaughlin M., Pedersen M., Roulstone V., Bergerhoff K.F., Smith H.G., Whittock H., Kyula J.N., Dillon M.T., Pandha H.S., Vile R. (2020). The PERK Inhibitor GSK2606414 Enhances Reovirus Infection in Head and Neck Squamous Cell Carcinoma via an ATF4-Dependent Mechanism. Mol. Ther. Oncolytics.

[80] Zweerink H.J., Joklik W.K. (1970). Studies on the intracellular synthesis of reovirus-specified proteins. Virology.

[81] Qin Q., Carroll K., Hastings C., Miller C.L. (2011). Mammalian orthoreovirus escape from host translational shutoff correlates with stress granule disruption and is independent of eIF2alpha phosphorylation and PKR. J. Virol..

[82] Choudhury P., Bussiere L.D., Miller C.L. (2017). Mammalian Orthoreovirus Factories Modulate Stress Granule Protein Localization by Interaction with G3BP1. J. Virol..

[83] Carroll K., Hastings C., Miller C.L. (2014). Amino acids 78 and 79 of Mammalian Orthoreovirus protein µNS are necessary for stress granule localization, core protein λ2 interaction, and de novo virus replication. Virology.

[84] Kedersha N., Panas M.D., Achorn C.A., Lyons S., Tisdale S., Hickman T., Thomas M., Lieberman J., McInerney G.M., Ivanov P. (2016). G3BP-Caprin1-USP10 complexes mediate stress granule condensation and associate with 40S subunits. J. Cell Biol..

[85] Huismans H., Joklik W.K. (1976). Reovirus-coded polypeptides in infected cells: Isolation of two native monomeric polypeptides with affinity for single-stranded and double-stranded RNA, respectively. Virology.

[86] Imani F., Jacobs B.L. (1988). Inhibitory activity for the interferon-induced protein kinase is associated with the reovirus serotype 1 sigma 3 protein. Proc. Natl. Acad. Sci. USA.

[87] Sidrauski C., Acosta-Alvear D., Khoutorsky A., Vedantham P., Hearn B.R., Li H., Gamache K., Gallagher C.M., Ang K.K., Wilson C. (2013). Pharmacological brake-release of mRNA translation enhances cognitive memory. Elife.

[88] Sidrauski C., McGeachy A.M., Ingolia N.T., Walter P. (2015). The small molecule ISRIB reverses the effects of eIF2α phosphorylation on translation and stress granule assembly. Elife.

[89] Tsai J.C., Miller-Vedam L.E., Anand A.A., Jaishankar P., Nguyen H.C., Renslo A.R., Frost A., Walter P. (2018). Structure of the nucleotide exchange factor eIF2B reveals mechanism of memory-enhancing molecule. Science.

[90] Rabouw H.H., Langereis M.A., Anand A.A., Visser L.J., de Groot R.J., Walter P., van Kuppeveld F.J.M. (2019). Small molecule ISRIB suppresses the integrated stress response within a defined window of activation. Proc. Natl. Acad. Sci. USA.

[91] Rabouw H.H., Visser L.J., Passchier T.C., Langereis M.A., Liu F., Giansanti P., van Vliet A.L.W., Dekker J.G., van der Grein S.G., Saucedo J.G. (2020). Inhibition of the integrated stress response by viral proteins that block p-eIF2-eIF2B association. Nat. Microbiol..

[92] Holcik M. (2015). Could the eIF2α-Independent Translation Be the Achilles Heel of Cancer?. Front. Oncol..

[93] Yamaguchi S., Ishihara H., Yamada T., Tamura A., Usui M., Tominaga R., Munakata Y., Satake C., Katagiri H., Tashiro F. (2008). ATF4-mediated induction of 4E-BP1 contributes to pancreatic beta cell survival under endoplasmic reticulum stress. Cell Metab..

[94] Mailliot J., Martin F. (2018). Viral internal ribosomal entry sites: Four classes for one goal. Wiley Interdiscip Rev. RNA.

[95] Jaafar Z.A., Kieft J.S. (2019). Viral RNA structure-based strategies to manipulate translation. Nat. Rev. Microbiol..

[96] Fernandez J., Yaman I., Sarnow P., Snider M.D., Hatzoglou M. (2002). Regulation of internal ribosomal entry site-mediated translation by phosphorylation of the translation initiation factor eIF2alpha. J. Biol. Chem..

[97] Terenin I.M., Dmitriev S.E., Andreev D.E., Shatsky I.N. (2008). Eukaryotic translation initiation machinery can operate in a bacterial-like mode without eIF2. Nat. Struct. Mol. Biol..

[98] White J.P., Reineke L.C., Lloyd R.E. (2011). Poliovirus switches to an eIF2-independent mode of translation during infection. J. Virol..

[99] Lee J.H., Choi S.K., Roll-Mecak A., Burley S.K., Dever T.E. (1999). Universal conservation in translation initiation revealed by human and archaeal homologs of bacterial translation initiation factor IF2. Proc. Natl. Acad. Sci. USA.

[100] Milon P., Carotti M., Konevega A.L., Wintermeyer W., Rodnina M.V., Gualerzi C.O. (2010). The ribosome-bound initiation factor 2 recruits initiator tRNA to the 30S initiation complex. EMBO Rep..

[101] Thakor N., Holcik M. (2012). IRES-mediated translation of cellular messenger RNA operates in eIF2α-independent manner during stress. Nucleic Acids Res..

[102] Yang Y., Wang Z. (2019). IRES-mediated cap-independent translation, a path leading to hidden proteome. J. Mol. Cell Biol..

[103] Haizel S.A., Bhardwaj U., Gonzalez R.L., Mitra S., Goss D.J. (2020). 5’-UTR recruitment of the translation initiation factor eIF4GI or DAP5 drives cap-independent translation of a subset of human mRNAs. J. Biol. Chem..

[104] Lewis S.M., Cerquozzi S., Graber T.E., Ungureanu N.H., Andrews M., Holcik M. (2008). The eIF4G homolog DAP5/p97 supports the translation of select mRNAs during endoplasmic reticulum stress. Nucleic Acids Res..

[105] Silvera D., Arju R., Darvishian F., Levine P.H., Zolfaghari L., Goldberg J., Hochman T., Formenti S.C., Schneider R.J. (2009). Essential role for eIF4GI overexpression in the pathogenesis of inflammatory breast cancer. Nat. Cell Biol..

[106] Skup D., Millward S. (1980). Reovirus-induced modification of cap-dependent translation in infected L cells. Proc. Natl. Acad. Sci. USA.

[107] Cho I.R., Koh S.S., Min H.J., Park E.H., Ratakorn S., Jhun B.H., Jeong S.H., Yoo Y.H., Youn H.D., Johnston R.N. (2010). Down-regulation of HIF-1alpha by oncolytic reovirus infection independently of VHL and p53. Cancer Gene Ther..

[108] Gupta-Saraf P., Miller C.L. (2014). HIF-1α downregulation and apoptosis in hypoxic prostate tumor cells infected with oncolytic mammalian orthoreovirus. Oncotarget.

[109] Kedersha N., Anderson P. (2007). Mammalian stress granules and processing bodies. Methods Enzymol..

[110] Jiang G., Santos Rocha C., Hirao L.A., Mendes E.A., Tang Y., Thompson G.R., Wong J.K., Dandekar S. (2017). HIV Exploits Antiviral Host Innate GCN2-ATF4 Signaling for Establishing Viral Replication Early in Infection. mBio.

[111] Qian Z., Xuan B., Chapa T.J., Gualberto N., Yu D. (2012). Murine cytomegalovirus targets transcription factor ATF4 to exploit the unfolded-protein response. J. Virol..

[112] Liang Q., Deng H., Sun C.W., Townes T.M., Zhu F. (2011). Negative regulation of IRF7 activation by activating transcription factor 4 suggests a cross-regulation between the IFN responses and the cellular integrated stress responses. J. Immunol..

[113] Connor J.H., Weiser D.C., Li S., Hallenbeck J.M., Shenolikar S. (2001). Growth arrest and DNA damage-inducible protein GADD34 assembles a novel signaling complex containing protein phosphatase 1 and inhibitor 1. Mol. Cell. Biol..

[114] Mroz E.A., Rocco J.W. (2017). The challenges of tumor genetic diversity. Cancer.

[115] Cheng T., Zhan X. (2017). Pattern recognition for predictive, preventive, and personalized medicine in cancer. EPMA J..

[116] Nangalia J., Campbell P.J. (2019). Genome Sequencing during a Patient’s Journey through Cancer. N. Engl. J. Med..

[117] Pazarentzos E., Bivona T.G. (2015). Adaptive stress signaling in targeted cancer therapy resistance. Oncogene.

[118] Grabocka E., Bar-Sagi D. (2016). Mutant KRAS Enhances Tumor Cell Fitness by Upregulating Stress Granules. Cell.

[119] Park Y.J., Choi D.W., Cho S.W., Han J., Yang S., Choi C.Y. (2020). Stress Granule Formation Attenuates RACK1-Mediated Apoptotic Cell Death Induced by Morusin. Int. J. Mol. Sci..

[120] Somasekharan S.P., El-Naggar A., Leprivier G., Cheng H., Hajee S., Grunewald T.G., Zhang F., Ng T., Delattre O., Evdokimova V. (2015). YB-1 regulates stress granule formation and tumor progression by translationally activating G3BP1. J. Cell Biol..

[121] Williams M.S., Amaral F.M., Simeoni F., Somervaille T.C. (2020). A stress-responsive enhancer induces dynamic drug resistance in acute myeloid leukemia. J. Clin. Investig..

[122] Kepp O., Semeraro M., Bravo-San Pedro J.M., Bloy N., Buqué A., Huang X., Zhou H., Senovilla L., Kroemer G., Galluzzi L. (2015). eIF2α phosphorylation as a biomarker of immunogenic cell death. Semin. Cancer Biol..

[123] Guo L., Chi Y., Xue J., Ma L., Shao Z., Wu J. (2017). Phosphorylated eIF2α predicts disease-free survival in triple-negative breast cancer patients. Sci. Rep..

[124] He Y., Correa A.M., Raso M.G., Hofstetter W.L., Fang B., Behrens C., Roth J.A., Zhou Y., Yu L., Wistuba I.I. (2011). The role of PKR/eIF2α signaling pathway in prognosis of non-small cell lung cancer. PLoS ONE.

